# *Mycobacterium tuberculosis* Latent Antigen Rv2029c from the Multistage DNA Vaccine A39 Drives TH1 Responses via TLR-mediated Macrophage Activation

**DOI:** 10.3389/fmicb.2017.02266

**Published:** 2017-11-17

**Authors:** Haibo Su, Shengling Zhu, Lin Zhu, Cong Kong, Qi Huang, Zhi Zhang, Honghai Wang, Ying Xu

**Affiliations:** ^1^State Key Laboratory of Genetic Engineering, Institute of Genetics, School of Life Science, Fudan University, Shanghai, China; ^2^GMU-GIBH Joint School of Life Science, Guangzhou Medical University and Guangzhou Institutes of Biomedicine and Health, Guangzhou, China; ^3^The Second People's Hospital of Guangdong Province, Guangzhou, China

**Keywords:** latent antigens, *Mycobacterium tuberculosis*, macrophages, tuberculosis, TB vaccine

## Abstract

Targeting of *Mycobacterium tuberculosis* (MTB) latent antigens comprises a crucial strategy for the development of alternative tuberculosis (TB) vaccine(s) that protects against TB reactivation. Here, we generated a multistage DNA vaccine, A39, containing the early antigens Ag85A and Rv3425 as well as the latency-associated protein Rv2029c, which conferred protective immunity in a pre-exposure mouse model. Moreover, administration of the A39 vaccination after MTB exposure inhibited reactivation and resulted in significantly lower bacterial loads in the lungs and spleen of mice, compared to those in the control population. Subsequently, we investigated the effect of Rv2029c on innate immunity and characterized the molecular details of the interaction of this protein with the host via iTRAQ proteomic and biochemical assay analyses. Rv2029c activated macrophages, triggered the production of pro-inflammatory cytokines, and promoted toll-like receptor/mitogen-activated protein kinase (TLR/MAPK)-dependent macrophage apoptosis. Furthermore, Rv2029c treatment enhanced the ability of *Mycobacterium bovis* Bacillus Calmette-Guérin (BCG)-infected macrophages to present antigens to CD4^+^ T cells *in vitro*, which correlated with an increase in MHC-II expression. Lastly, Rv2029c-treated macrophages activated T cells, effectively polarized CD4^+^ and CD8^+^ T cells to secrete IFN-γ and IL-2, and specifically expanded a population of CD44^high^CD62L^low^CD4^+^/CD8^+^ effector/memory cells, indicating that Rv2029c, as a specific recall antigen, contributes to Th1 polarization in T cell immunity. These results suggest that Rv2029c and A39 comprise promising targets for the development of next-generation clinical TB therapeutic vaccines.

## Introduction

*Mycobacterium tuberculosis* (MTB) is a human pathogen that has shown an outstanding ability to adapt to its host. Indeed, greater than one-third of the world's population is estimated to be infected with this organism, and millions of people succumb to MTB infection each year (O'Garra et al., 2013). Currently, the large global population of latently infected individuals results in high numbers of new active TB cases each year (Geluk et al., 2012). Treatment of TB involves a combination of chemical drugs, namely rifampicin, isoniazid, pyrazinamide, and ethambutol. However, administration of these drugs over a period of 6–9 months can lead to their non-compliance and to the development of multi-drug resistance (Lin et al., 2007). Due to the emergence of such multidrug-resistant strains, as well as increased numbers of individuals co-infected with MTB and the human immunodeficiency virus (HIV), the number latent MTB-infected persons is likely to soar in coming decades (Lin et al., 2007).

The live-attenuated *Mycobacterium bovis* Bacillus Calmette-Guérin (BCG) vaccine is currently the only vaccine for TB available. While this vaccine is effective in protecting against the severe childhood forms of tuberculosis (Trunz et al., 2006), it demonstrates variable efficacy against the pulmonary forms of TB in young adults, as well as in reactivated populations and in TB-endemic regions (Andersen and Doherty, 2005; Kaufmann and Gengenbacher, 2012). This could be explained, at least in part, by interference from environmental mycobacteria, and by the natural genetic variation present in both the host populations and the BCG vaccine strains used for vaccination (Brandt et al., 2002; Trunz et al., 2006; Lalor et al., 2009; Liu et al., 2009; Abdallah et al., 2015). The ineffectiveness of BCG, the non-compliance of TB drugs, and the emergence of individuals co-infected with HIV and MTB highlight the importance of the development of a new and improved vaccine that would protect the infected people against latency-causing MTB antigens (Singh et al., 2014). The ideal vaccines would drive immune responses toward recognizing multistage antigens expressed during the early/replication phase of MTB, as well as antigens expressed during latent infection or reactivation thereof Geluk et al. (2012).

Latency-associated antigens, such as those located in the dormancy survival regulon (DosR), are encoded by a set of MTB genes responsible for adaptation to latency, and have been shown to elicit strong immunogenic and protective responses (Ernst, 2012; Ottenhoff and Kaufmann, 2012; Singh et al., 2014). For example, the DosR-regulated protein HspX was found to be targeted by both CD4^+^/CD8^+^ T cells (Demissie et al., 2006). Moreover, a series of other latency proteins (Rv0079, Rv1737c, Rv2389c, Rv2031c, and Rv2628c) were shown to induce strong stimulation of cellular Th1-type immune responses (Kimuda et al., 2017). Indeed, a DNA vaccine consisting of eight DosR regulon-encoded antigens provided protection against MTB infection in BALB/c and C57BL/6 mice by inducing strong humoral and/or cellular Th1-type immune responses (Roupie et al., 2007). Additionally, rBCG overexpressing latency antigen Rv1733c showed superior protection to BCG in TB mouse models (Reece et al., 2011), while the multistage vaccine H56, consisting of Ag85A, ESAT-6, and Rv2660c (latency), boosted the protective effects of BCG against active tuberculosis and the reactivation of latent MTB infection (Aagaard et al., 2011; Lin et al., 2012). Given that this strategy is currently the most commonly utilized in the development of novel TB vaccines, a heterologous prime boost approach involving the incorporation of latent antigens to induce a Th1-specific cellular immune response would provide greater protection than a homologous prime boost approach (Zumla et al., 2016).

Macrophages recognize MTB cells, as well as subcellular fractions derived from MTB cells, through Toll-like receptors (TLRs). In particular, MTB and its products have been shown to trigger macrophage activation via TLR-2-dependent intracellular MyD88 signaling, resulting in the production of inflammatory cytokines and the expression of co-stimulatory molecules that promote a Th1-biased T cell response (Lopez et al., 2003). TLR12 is critical for the IRF8-dependent IL-12 production in response to bacterial infection. The production of reactive nitrogen intermediates in macrophages comprises one of the major defense mechanisms against infection by intracellular pathogens (Bogdan, 2001). Macrophages have also been shown to present MTB-specific antigens to the cell surface where they are recognized by T cells, leading to the initiation of an adaptive immune response. Thus, macrophages serve as the crucial antigen-presenting cell type that bridges the innate and adaptive immune systems, and are clearly involved in latent antigen-induced host immunity (Hmama et al., 2015).

Rv2029c (*pfkB*) is a member of the DOS regulon and is up-regulated during hypoxia and in macrophages (Shi et al., 2016). TraSH-based mutagenesis screening indicated that Rv2029c is not essential for *M. tuberculosis* growth *in vitro* or *in vivo* (Sassetti and Rubin, 2003; Phong et al., 2013). This protein was previously shown to function as a 6-phosphofructokinase (PfkB) that is involved in glycolysis, converting sugar-1-P to sugar-1, 6-P (Phong et al., 2013). DosR antigens Rv2029c of *M. tuberculosis* induced higher frequencies of CD4^+^ or CD8^+^ T cells producing interferon gamma (IFN-γ) and/or tumor necrosis alpha (TNF-α) in patients with long-term latent tuberculosis infection (ltLTBI), compared to those with pulmonary tuberculosis (PTB) (Riano et al., 2012; Araujo et al., 2015; Arroyo et al., 2016). Furthermore, this protein was predicted as possible vaccine candidate (Zvi et al., 2008). Thus, Rv2029c may function as a virulence determinant, and could therefore be used in vaccine development to confer protective immunity against MTB. In previous work, we demonstrated that the multistage A39 DNA vaccine, comprising antigens Ag85A, Rv3425, and Rv2029c (latency), elicited strong cell-mediated immune responses in mice (Song et al., 2015). In the present study, we build on these previous findings by evaluating the protective efficacy of A39 both before and after MTB exposure in mice. Moreover, we characterize the precise mechanism by which Rv2029c modulates host immunity by investigating the molecular features of the interactions between T cells and Rv2029c-presenting macrophages, and the subsequent memory T cell responses.

## Materials and methods

### Mice and cell lines

C57BL/6, TLR2 −/−, and TLR4 −/− mice were obtained from the Animal Center of Slaccas (Shanghai, China). All mice were housed under specific pathogen-free conditions in the Animal Center of the School of Life Science of Fudan University. All experimental procedures conformed to the Guidelines for the Care and Use of Laboratory Animals from the National Institutes of Health and were approved by the Animal Care and Use Ethical Committee of Fudan University. C57BL/6 mice were used for both the vaccination experiments and for the isolation of macrophages and T cells. TLR2 −/− and TLR4 −/− mice were used for TLR binding and pathway signaling assay analyses. Mouse peritoneal macrophages were isolated from TLR2−/− or C57BL/6 mice as previously described (Su et al., 2015). Briefly, the peritoneal cavities of euthanized mice were flushed with 5 ml of ice-cold RPMI 1640 medium without fetal bovine serum (FBS). Peritoneal cells were then enriched by centrifugation, seeded into six-well plates in RPMI 1640 containing 10% FBS, and incubated overnight at 37°C with 5% CO_2_. After removal of non-adherent cells, the remaining adherent cells were washed twice with PBS and treated with 10 μg/ml of Rv2029c for 24 h. RAW264.7 cells, which were used for both the iTRAQ mass spectrometry and immunological molecular biological assay analyses, were cultured in Dulbecco's modified Eagle's medium (DMEM) (Gibco, Grand Island, NY, USA) supplemented with 10% FBS, penicillin (100 U/ml), and streptomycin (100 mg/ml) at 37°C in a humidified incubator (5% CO_2_).

### Evaluation of the protective efficacy of the multistage A39 DNA before and after MTB exposure

**Figure 1:**
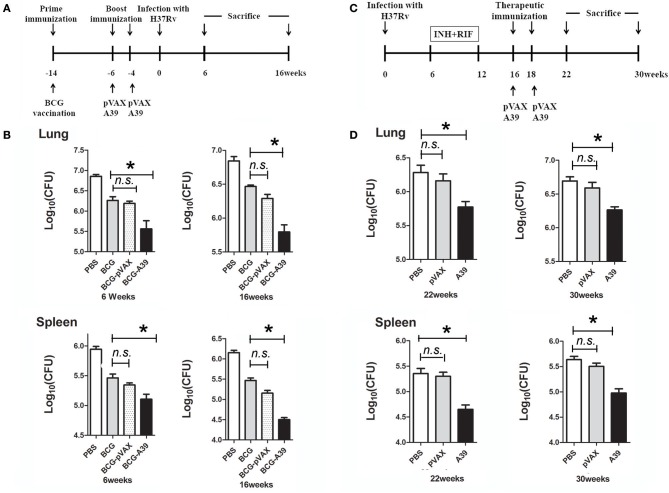
DNA vaccine A39 enhances the protective efficacy of the *Mycobacterium bovis* Bacillus Calmette-Guérin (BCG) vaccine both pre- and post-*Mycobacterium tuberculosis* (MTB) infection. **(A)** Timeline of animal vaccination, infection, and killing. Groups of C57BL/6 mice (*n* = 12 per group) were immunized subcutaneously with 5 × 10^6^ CFUs of BCG in 100 μl of PBS. At weeks 4 and 6 before infection, mice were boosted with 50 μg of pVAX (vector) or A39 (Ag85A-Rv3425-Rv2029c) via intramuscular injection in the right thigh. **(B)** At 4 weeks post-vaccination, all mice were aerosol challenged with 200 CFUs of MTB H37Rv. Then, at 6 and 16 weeks post-challenge, the mice were sacrificed, and the lungs and spleens were collected aseptically for quantification of the bacterial burden. **(C)** Flow chart depicting the post-exposure strategy. Mice were aerosol challenged with 5 × 10^3^ CFUs of MTB H37Rv. After 6 weeks, the mice were provided drinking water containing 0.1 g/l isoniazid (INH) and rifampicin (RIF) until week 12. On weeks 16 and 18 after infection, mice were treated with 50 μg of pVAX, or A39 via intramuscular injection in the right thigh. **(D)** The mice were sacrificed at 22 or 30 weeks post-challenge, and lungs and spleens were collected aseptically for enumeration of bacterial viable counts and histological evaluation (*n* = 6 mice per group). Data are presented as the means ± standard deviations of the results from two independent experiments. *P < 0.05.

Vaccination via the prime-boost strategy (Figure 1A) and the post-exposure strategy (Figure 1C) was conducted as previously described (Singh et al., 2014). All animals were divided into separate cages. For the prime-boost method, C57BL/6 mice (*n* = 12 per group) were immunized subcutaneously with 5 × 10^6^ CFUs of BCG in 100 μl of PBS. At weeks 4 and 6 prior to infection, mice were boosted with 50 μg pVAX (vector) or A39 (Ag85A-Rv3425-Rv2029c) via intramuscular injection in the right thigh. Then, at 4 weeks post-vaccination, all mice were aerosol challenged with 200 CFUs of MTB H37Rv in a 1-ml volume. Mice were sacrificed at 6 or 16 weeks post-challenge, and lungs and spleens were collected aseptically for histological evaluation and enumeration of viable bacteria.

For post-exposure analysis, C57BL/6 mice (*n* = 12 per group) were aerosol-challenged with 5 × 10^3^ CFUs of MTB. At 6 weeks post-challenge, the mice were provided drinking water containing 0.1 g/l isoniazid (INH) and 0.1 g/l rifampicin (RIF) per animal until week 12 for generation of the latent infection model. Then, on weeks 16 and 18 after infection, mice were administered 100 μl of 500 μg/ml pVAX (vector) or A39 (Ag85A-Rv3425-Rv2029c) via intramuscular injection in the right thigh. Mice were sacrificed at 22 or 30 weeks post-challenge, and the lungs and spleens were collected aseptically for histological evaluation and enumeration of viable bacteria.

### Cloning and expression of recombinant R2029c

The *R2029c* gene was amplified from MTB H37Rv genomic DNA by PCR using the following primers: Forward, 5′-ATTGAATTCATGACGGAGCCAGCGGCGT-3′; Reverse, 5′-ATTGCGGCCGC TCATGGCGAGGCTTCCGGGT-3′. The resulting PCR product was digested with the restriction enzymes EcoI and HindIII, subcloned into the expression vector pRSFDuet-1 (Novagene, Madison, WI, USA) and transformed into *Escherichia coli* BL21 (DE3) cells. Transformants were grown at 37°C to an optical density (OD) at 600 nm of 0.4 to 0.5, after which expression of the recombinant protein was induced by adding isopropyl-β-thiogalactopyranoside (IPTG) to a final concentration of 0.1 mM. After overnight cultivation at 15°C, the bacterial cells were harvested and lysed. Recombinant R2029c was then purified using HIS-Select® Nickel Affinity Gel (Sigma-Aldrich, St. Louis, MO, USA), as previously described but with minor modifications, dialyzed against PBS (pH 7.4), and treated with Pierce High Capacity Endotoxin Removal Resin (Pierce, Rockford, IL, USA), in accordance with the user instructions (Su et al., 2015). Endotoxin-free recombinant protein samples were quantified using a bicinchoninic acid (BCA) protein assay kit (Pierce) and frozen at −80°C, and recombinant Rv2029c expression was verified by western blot analysis using anti-R2029c-His mouse polyclonal anti-serum (Figure S4).

### Construction of rBCG-RV2029c

A pMV261 plasmid containing Rv2029c was introduced into BCG by electroporation. Selected rBCG-Rv2029c transformants were cultured in Middlebrook 7H9 medium containing 10% oleic albumin dextrose catalase (OADC) and 50 μg/ml kanamycin. The rBCG-Rv2029c strain was identified by western blot analysis using anti-Rv2029c-His mouse polyclonal anti-serum.

### Infection of macrophages and subcellular fractionation

BCG or rBCG-Rv2029c was cultivated in Middlebrook 7H9 medium containing 10% OADC until reaching exponential growth phase, after which samples were pelleted by centrifugation at 5,000 × g for 30 min. Prior to infection, a bacterial single-cell suspension was prepared by vortexing the cells with glass beads, followed by centrifugation at low speed and passaging through a 5-μm syringe filter to remove bacterial aggregates. RAW 264.7 cells (2 × 10^6^/well) were then infected at a multiplicity of infection (MOI) of 10 for 4 h at 37°C in a 5% CO_2_ environment. Cells were then harvested, and cytosolic and nuclear fractions were prepared using a NE-PER Nuclear and Cytoplasmic Extraction kit (Pierce). Briefly, similar amounts of BCG- or rBCG-Rv2029c-infected macrophages were suspended in buffer A containing a protease inhibitor cocktail (Sigma-Aldrich), incubated on ice for 10 min, and centrifuged at 5,000 × g at 4°C. The resulting supernatants were removed and the pelleted nuclear fractions were lysed for 30 min on ice in buffer B supplemented with a protease inhibitor cocktail (Sigma-Aldrich). The insoluble chromatin and the soluble nuclear protein were then separated by centrifugation at 12,000 × g for 15 min a 4°C. This procedure is summarized in Figure S1.

### iTRAQ labeling and strong cation exchange (SCX)-based fractionation

Approximately 200 μg of protein was dissolved in 50 mM TEAB (8 M Urea, 2% Triton X-100, and 0.1% SDS), reduced and alkylated via treatment with TCEP and the cysteine blocking agent MMTS, respectively, and digested with trypsin (Promega, Madison, WI, USA) at 37°C for 12 h. The digested peptides were then labeled using 4-plex iTRAQ reagent, according to the manufacturer's instructions (Applied Biosystems, Waltham, MA, USA) (Shadforth et al., 2005). SCX chromatography was performed as previously described (Shadforth et al., 2005). The peptides were fractionated on a PolySULFOETHYL A column (200 Å, 5 μm, 200 × 2.1 mm) (PolyLC, Columbia, MD, USA) using an Agilent 1200 LC system (Agilent Technologies, Santa Clara, CA, USA). Peptide fractions were collected using a linear gradient of solvent B (350 mM KCl in solvent A, pH 2.8) over 70 min at a flow rate of 300 μl/min. Subsequently, the pooled fractions were desalted using C18 stage tips (product number 2240/2340; 3M Empore, St. Paul, MN, USA) and analyzed using an LTQ Orbitrap XL ETD™ mass spectrometer (Thermo Fisher Scientific, Waltham, MA, USA).

### Bioinformatic analysis of identified proteins

Protein identification and quantification were carried out using Protein Discoverer 1.0 software and the MASCOT search engine. The parameters included for the searches were as follows: enzyme = trypsin; missed cleavage = 1; variable modification, oxidation (M); peptide mass tolerance = 20 ppm; mass spectrometry (MS)/MS tolerance = 0.1 Da; false discovery rate (FDR) ≤ 1%; iTRAQ modification at the N-terminus of the peptide and at lysine residues. The relative expression pattern of each protein was determined based on the relative intensities of the reporter ions for the corresponding peptides. *P*-values were computed using an online open-source quantitative proteomics *q*-value calculator (QPPC) tool. To elucidate the mechanism by which Rv2029c interacts with macrophages, we utilized the PANTHER (http://www.pantherdb.org/) and String (http://string.embl.de/) web tools, respectively, to cluster proteins associated with known signal cascades and to perform a data-dependent network analysis to map the possible connections between individual pathway components, signaling cascades, and concurrent signaling-modulated regulation for transcription.

### Measurement of cytokine expression levels

The levels of tumor necrosis factor (TNF)-α, interleukin (IL)-6, interferon (IFN)-γ, IL-4, IL-2, or IL-12p40 in the culture supernatants of macrophages or splenic T cells were determined using sandwich ELISA kits, according to the manufacturer's instructions (BioLegend, San Diego, CA, USA). For experiments designed to block toll-like receptor (TLR) signaling, RAW 264.7 cells (1 × 10^6^/well) were incubated for 1 h at 37°C with a mouse isotype IgG (30 μg/ml) or antibodies specific to TLR2 (30 μg/ml), TLR12 (30 μg/ml), TLR2 and TLR12 (30 μg/ml), or TLR4 (30 μg/ml) (all antibodies were purchased from BioLegend), respectively. For blockage of the mitogen-activated protein kinase (MAPK) signaling pathway, RAW 264.7 cells (1.0 × 10^6^/well) were pretreated with inhibitors of p38 (SB203580, 10 μM), ERK (U0126, 10 μM), or JNK (SP600125, 10 μM) for 1 h at 37°C, followed by incubation with R2029c for 36 h at 37°C. After 24 h of treatment, cytokine levels were measured by ELISA.

### Immunoprecipitation

RAW 264.7 cells (2.0 × 10^6^/well) were treated with Rv2029c (10 μg/ml) for 8 h and lysed with RIPA buffer (Sangon Biotech, Shanghai, China). The resulting lysates were precleared by incubating with protein A or G sepharose beads (Santa Cruz Biotechnology, Dallas, TX, USA) for 2 h. After centrifugation at 10,000 × *g* for 5 min at 4°C, supernatants were incubated with isotype IgG, anti-TLR2, anti-TLR12, or anti-Rv2029c antibodies overnight at 4°C. The beads were then harvested, washed, and boiled in 5 × sample buffer for 5 min. Proteins were separated via 10% SDS-PAGE and transferred electrophoretically to PVDF membranes (Millipore, Billerica, MA, USA). After washing with TBST (Tris-buffered saline containing 0.5% Tween-20) buffer, membranes were probed with anti-TLR2, anti-TLR12 (BioLegend), or anti-His (Santa Cruz Biotechnology) antibodies, as indicated, followed by horseradish peroxidase (HRP)-conjugated mouse anti-rat or rabbit anti-mouse IgG. Immunoreactive bands were detected using an enhanced chemiluminescent (ECL) reagent (Thermo Fisher Scientific) and visualized by exposure to x-ray film.

### Western blot analysis of kinase activation

RAW 264.7 cells (1.0 × 10^6^/well) were treated with medium, Rv2029c (10 μg/ml), lipopolysaccharide (LPS; 1 μg/ml), or Pam_3_CKS_4_ (Pam_3_CKS_4_ = Pam3, 5 μg /ml, a TLR1/2 agonist) for 1 h (Su et al., 2015), or infected with BCG or rBCG-R2029c for 0–8 h, then lysed in cell lysis buffer supplemented with a proteinase inhibitor mixture (Sigma-Aldrich). Cell pellets were processed using an NE-PER Nuclear and Cytoplasmic Extraction kit (Pierce), following the manufacturer's instructions for separation of the cytoplasmic and nuclear fractions. Equal amounts of proteins were separated by 10% SDS-PAGE and transferred electrophoretically to PVDF membranes (Millipore). Following blocking with 5% nonfat milk in TBST buffer, the membranes were incubated with the following primary antibodies overnight at 4°C: rabbit anti-ERK2, rabbit anti-p38, rabbit anti-JNK, rabbit anti-phospho (p)-ERK1/2, rabbit anti-p-p38, rabbit anti-p-JNK, rabbit anti-p-IκB-α, rabbit anti-NF-κB p65, rabbit anti-tubulin-α, or rabbit anti-β-actin (Santa Cruz Biotechnology), according to the supplier's instructions. After washing with TBST buffer, membranes were incubated with an HRP-conjugated secondary antibody for 1–2 h at 37°C. Target proteins were visualized using Pierce ECL Western Blotting Substrate (Thermo Fisher Scientific).

### Confocal microscopy analysis

RAW264.7 cells (1 × 10^6^/well) were seeded on coverslips and treated with Rv2029c for 2 h, after which they were fixed and permeabilized by treatment with cold methanol and 0.2% digitonin, respectively. After blocking with 2% BSA, cells were subjected to immunostaining by treatment with primary antibodies specific to p-p38, p-JNK, p-ERK1/2, p-IκBα, or NF-κB, for 4 h, followed by an AlexaFluor 488-conjugated secondary antibody for 2 h. Cells were then treated with 0.5 g/ml DAPI (Santa Cruz Biotechnology) for 5 min at 20°C for the visualization of nuclei. Coverslips were mounted onto slides using ProLong® Gold Antifade Mountant (Thermo Fisher Scientific) and observed using a 60 × oil objective lens on a Zeiss LSM 710 confocal laser microscope (Carl Zeiss, Oberkochen, Germany). Images were acquired using LSM710 Meta software and processed using ImageJ (1.4.4).

### TLR-binding assays

WT, TLR2−/−, and TLR4−/− peritoneal mouse macrophages isolated as described above were cultured overnight and then incubated with Rv2029c (10 μg/ml) for 2 h at 37°C. Following treatment, cells were fixed with 4% paraformaldehyde (PFA) for 15 min and permeabilized in 0.1% Triton X-100 (PBST) for 15 min. After blocking with 5% BSA in PBS containing 5% goat serum and 0.1% Tween-20 for 2 h, cells were treated with anti-TLR2 (1:200) and anti-His (1:500) antibodies overnight at 4°C, followed by an Alexa Fluor®568-conjugated donkey anti-mouse IgG (Santa Cruz Biotechnology) or an Alexa Fluor®488-conjugated donkey anti-rabbit IgG (Santa Cruz Biotechnology) secondary antibody for 2 h in the dark. Cells were then stained with 0.5 g/ml DAPI (Santa Cruz Biotechnology) for 5 min at room temperature. After washing, the cells were mounted onto slides using ProLong® Gold Antifade Mountant (Thermo Fisher Scientific) and observed using a 60 × oil objective on a Zeiss LSM 710 confocal laser microscope (Carl Zeiss). Images were acquired using LSM710Meta software and processed using image J (1.4.4).

### Flow cytometric analysis of MHC II expression

After prepared as described above, Mouse peritoneal macrophages (1 × 10^6^/well) were incubated with isotype IgG (30 μg/ml), anti-TLR2 antibodies (30 μg/ml), or anti-TLR2 and anti-TLR12 (30 μg/ml) antibodies for 18 h, followed by treatment with medium, LPS (positive control, 1 μg/ml) or Rv2029c (10 μg/ml) for 24 h, respectively. Following treatment, cells were harvested and washed with pre-chilled PBS and collected by centrifugation at 1,000 × *g* for 10 min at 4°C. Macrophages were then treated with Fc Block (1:100) (BD Pharmingen, San Jose, CA, USA) diluted in PBS supplemented with 1% BSA, and the expression levels of MHC II proteins were evaluated by staining with PE-conjugated anti-mouse CD86, FITC-conjugated anti-mouse CD80, PE-conjugated anti-mouse H-2κB for mouse macrophages (BD Pharmingen), or FITC-conjugated anti-mouse I-A/I-E antibodies on ice for 1 h in the dark. In addition, to examine the intracellular expression levels of IL-12p70 and IL-10, mouse peritoneal macrophages were treated with specific FITC- and PE-conjugated monoclonal antibodies, respectively. Cells were then evaluated by flow cytometry (Becton Dickinson, USA). Lastly, for antigen presentation assay, macrophages were treated for 18 h with isotype IgG (30 μg/ml), anti-TLR2 (30 μg/ml), or anti-TLR2 and anti-TLR12 (30 μg/ml) antibodies, followed by Rv2029c (10 μg/ml) or Pam_3_CKS_4_ (5 μg /ml) overnight. Following treatment, the cells were infected with BCG for 4 h, and the expression levels of MHC molecules were evaluated by flow cytometry using specific antibodies. The resulting data were analyzed using CellQuest data analysis software and FlowJo 10.0 software (Tree Star, Inc., Ashland, OR, USA).

### Flow cytometric analysis of cell apoptosis

Mouse peritoneal macrophageswere infected with 1.5 × 10^6^ CFUs of wild-type BCG, rBCG-pMV261, or rBCG-Rv2029c (MOI = 10) for 18 h or 36 h, or were pre-treated with isotype IgG (30 μg/ml), anti-TLR2 (30 μg/ml), anti-TLR12 (30 μg/ml), anti-TLR2 and anti-TLR12 (30 μg/ml), or anti-TLR4 (30 μg/ml) antibodies for 18 h, followed by infection with wild-type BCG, rBCG-pMV261 or rBCG-Rv2029c for 48 h (MOI = 10). Cells were then stained with an Annexin-V-conjugated dye and propidium iodide (PI) from an Apoptosis Detection Kit I (BD Pharmingen), and screened for apoptosis via flow cytometry analysis using a BD FACSCanto II device (BD Biosciences). The resulting data were analyzed with FlowJo 10.0 software (Tree Star, Inc.). All experiments were performed in biological triplicate, and the presented data are representative of at least three independent experiments.

### Analysis of the Th1 response and T-cell proliferation assay

T cells were isolated from total mononuclear cells of C57BL/6 mice immunized with Rv2029c (50 μg) using a magnetic activated cell sorting (MACS) column. The purity of the T cell populations isolated through MACS was >85%, as determined by flow cytometry analysis (BD Biosciences). Meanwhile, mouse peritoneal macrophages from C57BL/6 mice were treated with Rv2029c (10 μg/ml) or Pam_3_CKS_4_ (5 μg/ml) for 24 h. Mouse peritoneal macrophages were isolated from TLR2−/− or C57BL/6 mice as described above.

T cells and mouse macrophages were then co-cultured at a ratio of 10:1 for 3 days at 37°C and then pulsed with an anti-CD3e (5 μg/ml) antibody. T cells alone and T cells co-cultured with untreated macrophages served as controls. Sandwich ELISA kits were used to detect the levels of IFN-γ, IL-4, and IL-2 in the resulting culture supernatants, per the manufacturer's instructions (BioLegend). Additionally, harvested cells were stained with FITC-conjugated anti-CD4, PE-conjugated anti-chemokine receptor 3 (CCR3), or PE-conjugated anti-CXC chemokine receptor 3 (CXCR3) monoclonal antibodies (BD Pharmingen), and T cell proliferation was assessed by flow cytometry analysis (BD Biosciences). Lastly, to evaluate the activation of effector/memory T cells, C57BL/6 mice were immunized three times, at 2-week intervals, via subcutaneous injection of 5 × 10^6^ CFUs BCG or rBCG-Rv2029c in 100 μl of PBS. Lymphocytes were then isolated from total splenic cells at 12 weeks post-vaccination via Lymphocyte-M density-gradient centrifugation, after which the resulting macrophages were stimulated with Rv2029c (10 μg/ml), Pam_3_CKS_4_ (5 μg/ml), or PBS (control) for 24 h and co-cultured with T cells at a ratio of 1:10 for 3 days. Cells were then stained with PE-Cy5-conjugated anti-CD4 or PE-Cy5-conjugated anti-CD8 monoclonal antibodies, and FITC-conjugated anti-CD62L and PE-conjugated anti-CD44 monoclonal antibodies, and analyzed by flow cytometry.

### Statistical analysis

Results are presented as the means ± standard deviations (SD) of the results obtained from triplicate experiments. Results were analyzed by one-way analysis of variance (ANOVA) followed by Tukey's test or Dunnet's test, using Origin 8.0 software (Origin Lab, Northampton, MA, USA). For all tests, *P* ≤ 0.05 was considered statistically significant.

## Results

### Rv2029c, a key component of the A39 multistage DNA vaccine, confers protective immunity against MTB

The DNA vaccine A39 contains MTB antigens Ag85A and Rv3425, both of which are expressed during the early and acute phases of infection, and the hypoxia stress-induced antigen Rv2029c. Here, we evaluated the protective efficacy of boosting with A39 after immunization with BCG (BCG prime-A39 boost) both before and after challenge with live MTB H37Rv (Figure 1A). For these experiments, the bacterial loads in the lungs and spleens of mice were analyzed at 6 and 16 weeks post-challenge. All mice in the BCG-vaccinated groups exhibited significantly fewer bacteria in the lungs and spleens than those in the PBS control group. The BCG prime-A39 boost strategy provided the greatest protection against MTB, with significantly lower CFUs in the lungs and spleens after challenge than those in the BCG after challenge group. In addition, the CFU numbers in the BCG prime-A39 boost-immunized groups were lower than those in the BCG prime-pVAX boost group (Figure 1B). Together, these results indicate that boosting BCG with A39 results in enhanced control of bacterial replication in the lungs and spleens of MTB-infected mice.

The observed protective efficacy of A39 encouraged us to test the ability of this vaccine to control reactivation of MTB infection. As a model for post-exposure vaccination, C57BL mice were infected via aerosol exposure and then treated with INH and RIF, or with the pVAX or A39 DNA vaccines (Figure 1C). While the bacterial load post-antibiotic treatment was low, robust reactivation was observed at 22 weeks after infection. Conversely, as shown in Figure 1D, the lungs and spleens of A39-immunized mice exhibited significantly lower numbers of MTB than those in the pVAX and PBS control groups (0.5–0.7 log10 reduction in CFUs), even at 22 weeks post-infection. These results demonstrate that A39 provides a significant degree of protection against reactivation of MTB infection in mice.

### Rv2029c induces an inflammatory response via TLR2/TLR12-mediated cross-talk among activated downstream pathways in mouse macrophages

**Figure 2:**
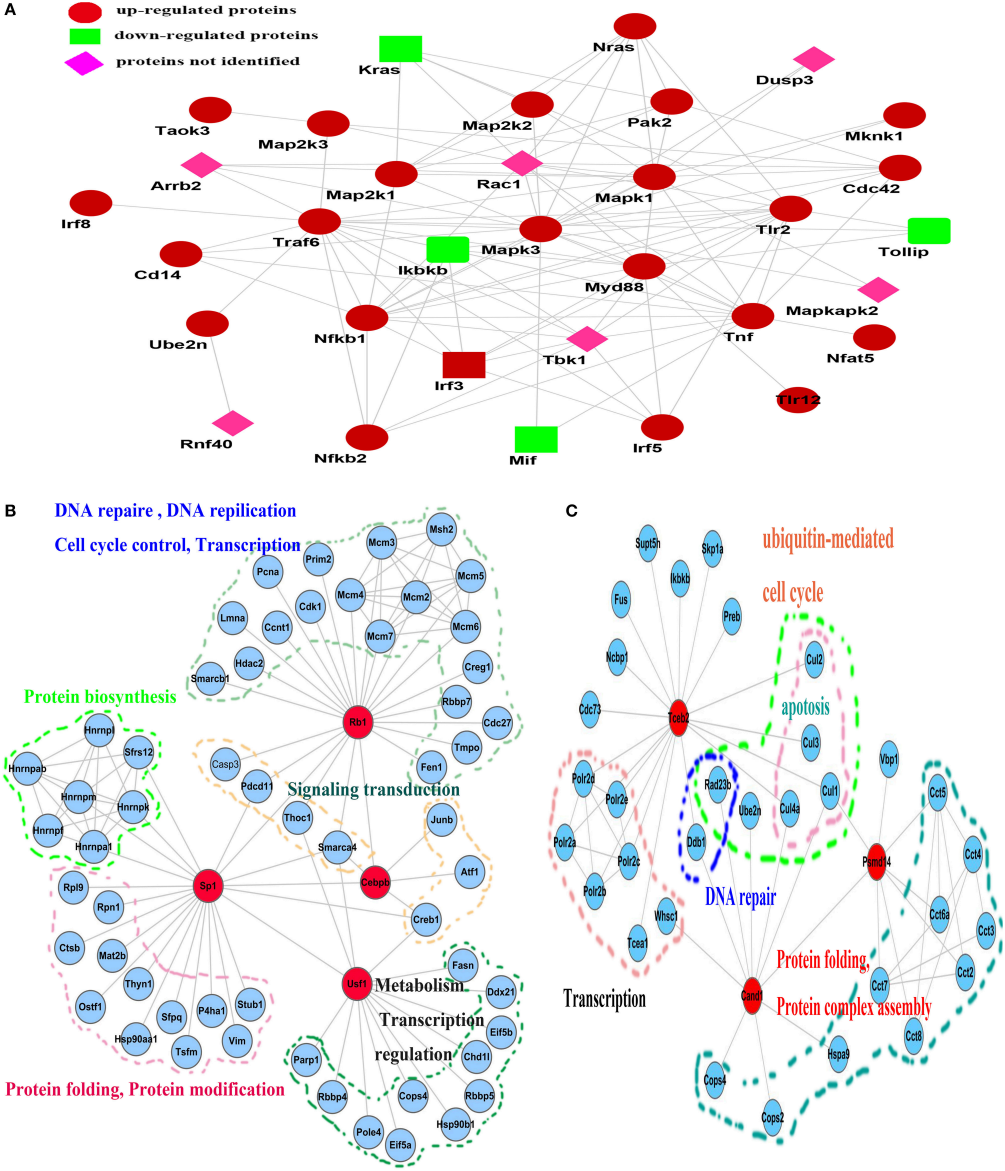
Reconstruction of the network, involving multiple signaling cascades, activated in response to Rv2029c stimulation in RAW264.7 macrophages. **(A)** Key components whose H/L ratios were >1.2 or <0.8 were acquired by PANTHER (http://www.pantherdb.org/) and then submitted to STRING (http://string.embl.de/) for network construction. Cross-talk among the TLR2/TLR12, NF-κB, MAPK, and interferon regulatory factor (IRF) signaling pathways was interconnected with transcription factors and transcription factor-associated proteins to regulate the inflammatory response. **(B,C)** Subnetworks identified by data-dependent network analysis and their functional links to different biological processes. **(B)** The subnetwork involving Rb1, Sp1, Usf1, Cebpb, and their associated proteins; **(C)** the subnetwork involving Tceb2, Cand1, and Psmd14. The network was edited using Cytospace 3.1.0 software.

To clarify the mechanism by which Rv2029c influences host immunity, we designed an iTRAQ-based subcellular quantitative proteomic approach to identify proteins associated with Rv2029c functions in BCG- or rBCG-Rv2029c-infected RAW264.7 cells (Figure S1). Via these analyses, we found that proteins exhibiting differential expression in response to Rv2029c were primarily associated with signal transduction, immunology and defense, response to stress, and apoptosis (Tables S1, S2, and Figure S2). As shown in Figure 2A, dynamic linkages involving the key components of the TLR2/TLR12, MAPKs, NF-κB, and IRF signaling pathways may coordinate the Rv2029c-triggered extensive inflammatory response observed in MTB-infected macrophages. Moreover, the detailed sub-networks of global functional links among transcription factors, cofactors, and their associated proteins elucidates how the transcription factor regulatory network operates in an interconnected manner with upstream signaling cascades during Rv2029c challenge (Figures 2B,C).

### Rv2029c promotes NF-κB, Jnk, p38, and ERK1/2 activation in macrophages

**Figure 3:**
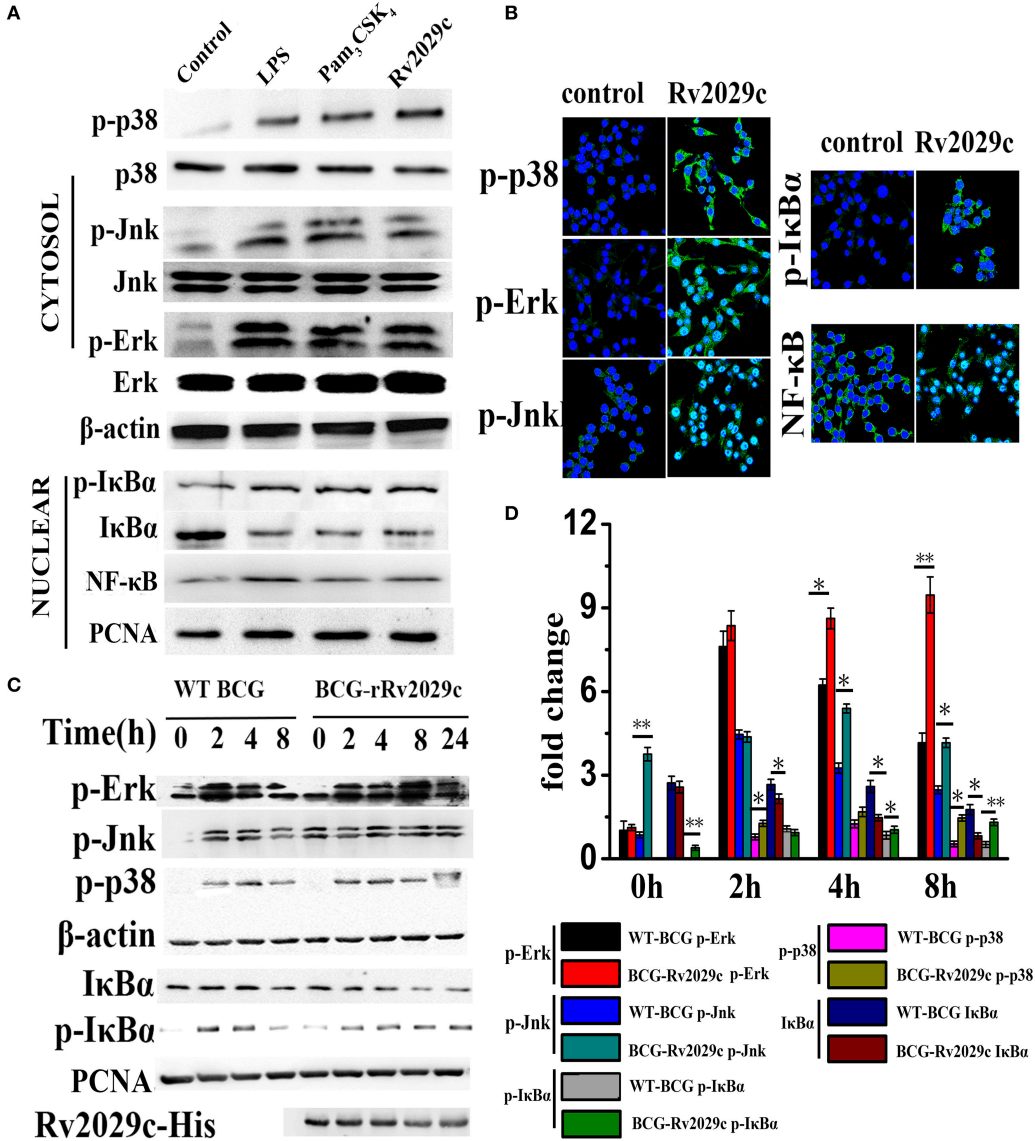
*Mycobacterium tuberculosis* (MTB) antigen Rv2029c promotes NF-κB and MAPK pathway activation. **(A)** The phosphorylation status of NF-κB, p38, ERK (1/2), JNK, and IκBα, as well as the nuclear translocation of NF-κB, in RAW264.7 cells treated with medium, Rv2029c (10 μg/ml), LPS (1 μg/ml), or Pam_3_CKS_4_ (Pam3, 5 μg/ml) for 1 h were evaluated by western blot analysis. **(B)** Immunofluorescence microscopy analysis of the levels of phosphorylated p38, JNK, ERK1/2, and IκBα, and of nuclear translocation of NF-κB in mouse primary macrophages treated with Rv2029c for 2 h. Scale bar, 50 μm. **(C)** Immunoblot analysis of the levels of phosphorylated NF-κB, p38, ERK (1/2), JNK, and IκBα, and of nuclear translocated NF-κB in RAW264.7 cells infected for 0–8 h with 2 × 10^6^ CFUs of wild-type *Mycobacterium bovis* Bacillus Calmette-Guérin (BCG) or rBCG-Rv2029c. **(D)** Densitometric analysis of the bands present in the western blot shown in panel **(C)**, presented relative to β-actin. All data are expressed as the means ± standard deviations of the results obtained from three separate experiments. ^*^P < 0.05; ^**^P < 0.01.

To further investigate whether Rv2029c activates the MAPK and NF-κB signaling pathways, as indicated by MS data, RAW264.7 cells were treated with culture medium, Rv2029c (10 μg/ml), LPS (1 μg/ml), or Pam_3_CKS_4_ (5 μg/ml) for various lengths of time, and the phosphorylation status of p38, JNK, and ERK1/2 was evaluated by western blot analysis. As shown in Figure 3A, stimulation with Rv2029c for 1 h triggered strong phosphorylation of p38, JNK, and ERK1/2. Conversely, little phosphorylation was observed in untreated cells. Similar results were observed by confocal microscopy analysis of cells stained with antibodies specific to p-p38, p-JNK, and p-ERK1/2 (Figure 3B).

Next, we examined the intracellular localization of NF-κB subunits in RAW264.7 cells treated with or without Rv2029c for 1 h. Treatment with Rv2029c resulted in increased nuclear translocation of NF-κB, and a corresponding decrease in the nuclear expression of Iκ-Bα (the inhibitor of NF-κB), compared to the control cells, as determined by western blot analysis (Figure 3A). Again, these findings were supported by confocal microscopy analysis (Figure 3B).

Lastly, we investigated the effects of Rv2029c on NF-κB and MAPK activation in BCG- or rBCG-Rv2029c-infected RAW264.7 cells. As shown in Figures 3C,D, rBCG-Rv2029c infection resulted in enhanced phosphorylation of p38, JNK, and ERK1/2, as well as increased nuclear translocation of NF-κB, compared to infection with wild type BCG, at 4 h post-infection. These data were inconsistent with those obtained by MS, indicating that Rv2029c could activate the innate immune system via activation of the NF-κB, JNK, ERK1/2, and p38 signaling pathways in macrophages.

### Rv2029c induces pro-inflammatory cytokine production in macrophages in a TLR2/TLR12/MAPK pathway-dependent manner

**Figure 4:**
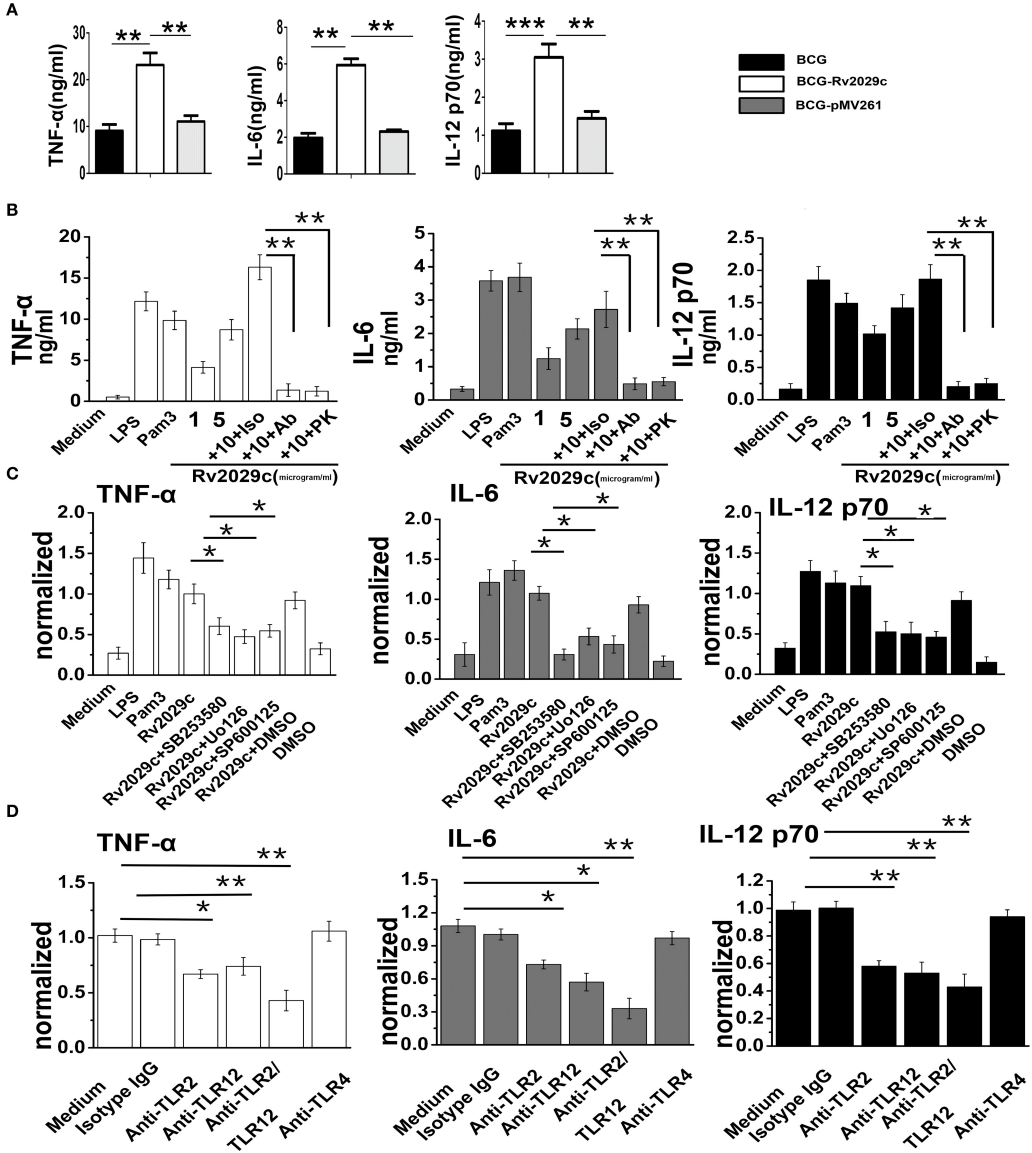
*Mycobacterium tuberculosis* (MTB) antigen Rv2029c activates macrophages via a MAPK and TLR2/TLR12 pathway-dependent mechanism. **(A)** ELISA analysis of TNF-α, IL-6, and IL-12 p70 production in RAW264.7 cells infected for 24 h with *Mycobacterium bovis* Bacillus Calmette-Guérin (BCG), rBCG-pMV261, or rBCG-Rv2029c. **(B)** RAW264.7 cells were incubated with Rv2029c (10 μg/ml), proteinase K (PK, 50 mg/ml), Pam3 (5 μg/ml), or LPS (1 μg/ml) for 36 h, after which the levels of TNF-α, IL-6, and IL-12 p70 production were evaluated by ELISA. **(C)** RAW264.7 cells were treated with DMSO (vehicle control) or with inhibitors of p38 (SB203580, 10 μM), ERK (U0126, 10 μM), or JNK (SP600125, 10 μM) for 1 h at 37°C, then stimulated with Rv2029c (10 μg/ml) for 36 h. The levels of TNF-α, IL-6, and IL-12 p70 in the supernatants harvested from cells in each group were then assessed by ELISA. **(D)** ELISA analysis of the levels of TNF-α, IL-6, and IL-12 p70 production by RAW264.7 cells treated with isotype IgG (30 μg/ml), anti-TLR2 (30 μg/ml), anti-TLR12 (30 μg/ml), anti-TLR2 and anti-TLR12 (30 μg/ml), or anti-TLR4 (30 μg/ml) antibodies for 1 h, followed by Rv2029c (20 μg/ml) for 36 h. All data are expressed as the means ± standard deviations of the results obtained from three separate experiments. n.s., not significant; ^*^P < 0.05; ^**^P < 0.01.

Inflammatory cytokines are essential for the activation and recruitment of immune cells to sites of bacterial infection. We therefore examined whether Rv2029c influences the expression of the pro-inflammatory cytokines TNF-α, IL-6, and IL-12p70, and of the anti-inflammatory cytokine IL-10, in RAW264.7 infected with BCG, BCG-pMV261, or rBCG-Rv2029c, respectively, via ELISA analysis. After 24 h of infection, cells infected with the Rv2029c-overexpressing BCG exhibited markedly higher expression of TNF-α and IL-6 than those infected with BCG or BCG-pMV261. Meanwhile, there were no differences between groups in the levels of IL-10 production (Figure 4A and Figure S3). Together, these data indicate that Rv2029c induces a pro-inflammatory cytokine response. Additionally, we evaluated the levels of cytokine production in macrophages treated with various concentrations of recombinant Rv2029c (1–10 μg/ml), or with LPS (1 μg/ml), Pam_3_CKS_4_ (5 μg/ml), isotype antibodies (Iso, 50 μg/ml), anti-Rv2029c antibodies (50 μg/ml), or proteinase K (PK, 50 μg/ml) for 24 h by ELISA analysis. Treatment with Rv2029c resulted in a significant, dose-dependent increase in TNF-α, IL-6, and IL-12p70 production. Moreover, these effects were specific to Rv2029c, as the observed increases in these cytokines were abrogated by treatment with proteinase K or anti-Rv2029c, but not with the isotype control antibody (Figure 4B).

Our MS data indicated that Rv2029c induced the activation of MAPK pathways, which are essential for triggering the inflammatory response. We therefore examined whether MAPK signaling was involved in Rv2029c-induced pro-inflammatory cytokine production. Specifically, RAW264.7 cells were pretreated with a p38 inhibitor (SB203580, 30 μM), an ERK1/2 inhibitor (U0126, 50 μM), or a JNK inhibitor (SP600125, 50 μM) for 1 h prior to stimulation with Rv2029c (10 μg/ml) for 24 h, after which the levels of TNF-α, IL-6, and IL-12p70 production were measured by ELISA analysis. Predictably, treatment with the MAPK inhibitors resulted in significant abrogation of TNF-α, IL-6, and IL-12p70 production by Rv2029c-treated cells, indicating that these pathways are critical for MTB-mediated induction of pro-inflammatory cytokines (Figure 4C).

To investigate the roles of TLR2/TLR12 in Rv2029c induced-cytokine production, RAW264.7 cells were treated with or without anti-TLR2, anti-TLR12, anti-TLR4, or isotype control antibodies for 1 h, then stimulated with Rv2029c (10 μg/ml) for 24 h. The culture supernatants were then harvested, and the levels of TNF-α, IL-6, and IL-12p70 were measured by ELISA. As shown in Figure 4D, treatment with the anti-TLR2 and anti-TLR12 antibodies, respectively, but not the anti-TLR4 or isotype control antibodies, significantly blocked the observed Rv2029c-induced increases in TNF-α, IL-6, and IL-12p70 production. Moreover, immunoprecipitation analysis demonstrated that Rv2029c is capable of directly binding to TLR2, but not to TLR12 (Figure S4), indicating that Rv2029c induces macrophage activation and promotes pro-inflammatory cytokine production in a TLR2/MAPK signaling-dependent manner.

### Rv2029c challenge induces apoptosis in BCG-infected macrophages

**Figure 5:**
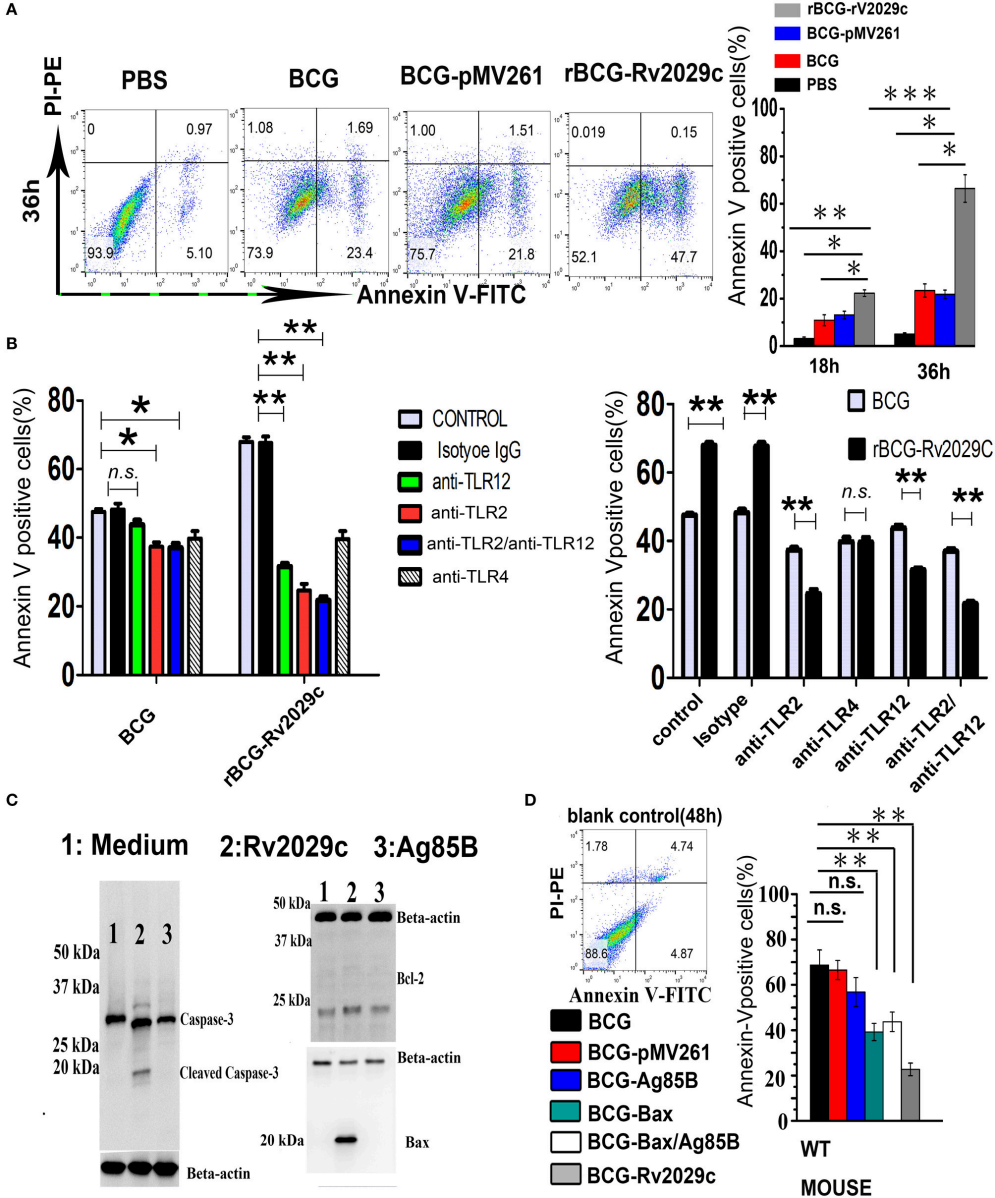
Over-expression of the *Mycobacterium tuberculosis* antigen Rv2029c in BCG regulates macrophage apoptosis dependent on TLR2/TLR12. **(A,B)** Graphic depictions of the numbers of apoptotic macrophages among mouse peritoneal macrophages (1.5 × 10^5^) **(A)** infected with 1.5 × 10^6^ CFUs of wild-type BCG, rBCG-pMV261, or rBCG-Rv2029c for 18 or 36 h (MOI = 1:10) or **(B)** treated with isotype IgG (30 μg/ml), anti-TLR2 (30 μg/ml), anti-TLR12 (30 μg/ml), anti-TLR2 and anti-TLR12 (30 μg/ml), or anti-TLR4 (30 μg/ml) antibodies for 18 h, then infected with wild-type BCG, or rBCG-Rv2029c for 48 h (MOI = 1:10). Following treatment all cells were stained with FITC-conjugated Annexin-V and PE-conjugated PI. **(C)** Western blot analysis of the levels of Bcl-2, Bax, and caspase-3 expression in mouse peritoneal macrophages infected with BCG or rBCG-Rv2029c for 72 h (MOI = 1:10). **(D)** Mice were immunized with BCG, BCG-pMV261 (vector control), BCG-Ag85B, BCG-Bax (positive control), BCG-Bax/Ag85B (positive control), or BCG-Rv2029c. The levels of apoptosis among alveolar macrophages were then determined by flow cytometry at 12 weeks after immunization. All data are expressed as the means ± standard deviations of the results obtained from three separate experiments. n.s., not significant; ^*^P < 0.05; ^**^P < 0.01.

MTB is capable of evading the host immune response by inhibiting macrophage apoptosis, which was previously shown to contribute to the extermination of intracellular pathogens (Behar et al., 2011). Via MS analysis (Table S3), we found that apoptosis-associated proteins such as Bax, Caspase 3, and Mlh2 were differentially expressed following Rv2029c challenge, and that this antigen triggered an apoptotic cross-talk network (Figure S5). We therefore analyzed the levels of apoptosis exhibited by Mouse peritoneal macrophages at 36 h after infection with BCG, rBCG-pMV261, or rBCG-Rv2029c by flow cytometry. As shown in Figure 5A, the apoptosis rate of macrophages infected with the Rv2029c-overexpressing BCG strain was significantly higher than that of the cells infected with wild-type bacteria.

TLR engagement elicits an array of cellular reprogramming genes, culminating in the apoptosis of infected macrophages. To investigate whether TLR2/TLR12 is involved in Rv2029c-enhanced apoptosis in BCG-infected macrophages, Mouse peritoneal macrophages were treated with isotype IgG (30 μg/ml), anti-TLR2 (30 μg/ml), anti-TLR12 (30 μg/ml), anti-TLR2 (30 μg/ml) + anti-TLR12 (30 μg/ml), or anti-TLR4 (30 μg/ml) antibodies for 18 h and then infected with BCG, or rBCG-Rv2029c for 48 h, after which the levels of apoptosis were analyzed by flow cytometry. Treatment with anti-TLR2 and anti-TLR12 antibodies, but not with the anti-TLR4 or the isotype-matched control antibodies, abrogated the rBCG-Rv2029c-induced apoptosis observed in mouse macrophages, suggesting that Rv2029c enhanced BCG-mediated apoptosis in macrophage via TLR2/TLR12-dependent manner (Figure 5B).

To further evaluate the pro-apoptotic effects of Rv2029c, RAW264.7 cells were treated with medium, Ag85B (10 μg/ml), or Rv2029c (10 μg/ml) for 24 h, after which the expression levels of Bcl-2, Bax, and caspase-3 were determined by western blot analysis. Consistent with its role in inducing apoptosis, Rv2029c treatment significantly promoted the expression of Bax and caspase-3, but not Bcl-2, compared to that observed in the other groups (Figure 5C). Next, we examined whether Rv2029c promotes BCG-mediated apoptosis in macrophage *in vivo*. For these studies, mice were immunized with BCG, BCG-pMV261 (vector control), BCG-Ag85B, BCG-Bax (positive control), BCG-Bax/Ag85B (positive control), or rBCG-Rv2029c. The levels of apoptosis among peritoneal macrophages were then detected by flow cytometry at 12 weeks after immunization. As shown in Figure 5D, the apoptosis rate was higher among the mice in the rBCG-Rv2029c group than in those of the BCG-Bax and BCG-Bax/Ag85B groups. Meanwhile, no significant changes in macrophages apoptosis were observed among the mice in the BCG, BCG-pMV261, or BCG-Ag85B groups. These results indicate that Rv2029c may trigger both apoptotic pathways to promoted BCG-induced apoptosis in macrophage.

### Rv2029c reverses the BCG-mediated reduction in MHC-II expression of mouse macrophages via its TLR2/TLR12 activation

**Figure 6:**
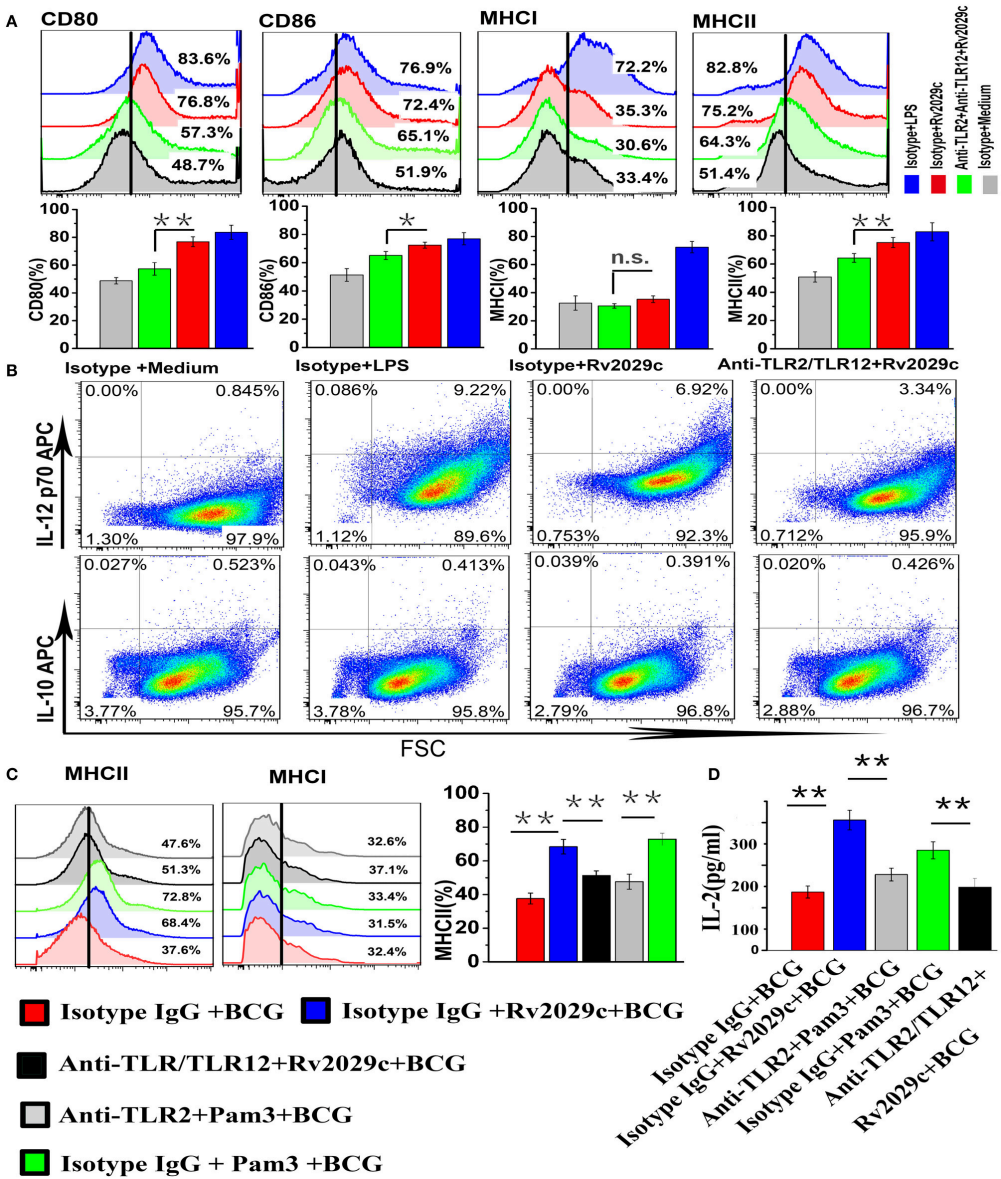
Rv2029c-induced activation of TLR2/TLR12 enhances macrophage function and increases MHC-II expression in BCG-infected macrophages. **(A)** Flow cytometry analysis of the expression of the cell surface markers CD80, CD86, MHC I, and MHC II in mouse peritoneal macrophages incubated with isotype IgG (30 μg/ml) or anti-TLR2 and anti-TLR12 (30 μg/ml) antibodies for 18 h, then stimulated with culture medium, LPS (positive control, 1 μg/ml), or Rv2029c (10 μg/ml) for 24 h. Cells were stained with FITC- or PE-conjugated monoclonal antibodies specific to the respective markers. **(B)** Flow cytometry analysis of the levels of intracellular IL-12p70 and IL-10 expression in mouse peritoneal macrophages incubated with isotype IgG (30 μg/ml) or anti-TLR2 and anti-TLR12 (30 μg/ml) antibodies for 18 h, then stimulated with culture medium, LPS (positive control, 1 μg/ml), or Rv2029c (10 μg/ml) for24 h. **(C)** Flow cytometry analysis of the expression of MHC molecules in mouse peritoneal macrophages treated with isotype IgG (30 μg/ml), anti-TLR2 (30 μg/ml), or anti-TLR2 and anti-TLR12 (30 μg/ml) antibodies for 18 h, then stimulated with Rv2029c (10 μg/ml) or Pam3 (5 μg /ml) overnight, infected with BCG for 4 h, washed, and overlaid with Rv2029c-specific T cells (1:10). **(D)** Graphic depiction of the levels of IL-2 in the supernatants of the cells described in **(C)**, as measured by sandwich ELISA analysis after 24 h. All data are expressed as the means ± standard deviations of the results obtained from three separate experiments. n.s., not significant; ^*^P < 0.05; ^**^P < 0.01.

To investigate the effect of Rv2029c-induced TLR2/TLR12 activation on macrophage function, mouse peritoneal macrophages were stimulated with culture medium, LPS (1 μg/ml), or Rv2029c (10 μg/ml) for 48 h after incubation with isotype IgG or with anti-TLR2 and anti-TLR12 antibodies, and the expression of cell surface markers, including CD80, CD86, MHC I, and MHC II, was examined by flow cytometry. Rv2029c treatment significantly increased the expression of CD80, CD86, and MHC II; in contrast, there was no change in MHC I expression (Figure 6). Meanwhile, blocking with anti-TLR2/anti-TLR12 antibodies abrogated the Rv2029c-mediated increases in CD80, CD86, and MHC II expression. We then investigated the levels of IL-12p70 and IL-10 production induced by Rv2029c stimulation, which are associated with the development of Th1 and Th2 cells, respectively. Rv2029c significantly induced the secretion of IL-12p70 but not of IL-10 (Figure 6B), and this effect was inhibited by treatment with anti-TLR2/anti-TLR12 antibodies, indicating that Rv2029c enhances macrophage function through TLR2/TLR12.

TLR activation was critical for the enhanced MHC-II expression relative to BCG-infected macrophages. To establish whether Rv2029c-mediated TLR2/TLR12 activation reversed the reduction of MHC-II expression induced by BCG infection, mouse peritoneal macrophages were treated with isotype IgG, isotype IgG and Pam3, isotype IgG and Rv2029c, anti-TLR2 antibodies and Pam3, or anti-TLR2 and anti-TLR12 antibodies and Rv2029c for 24 h, infected with BCG, and subjected to flow cytometry analysis to evaluate the surface expression of MHC-II. Additionally, the remaining macrophages were incubated with Rv2029c-specific T cells (1:10) for 24 h, after which the supernatants were collected and subjected to sandwich ELISA analysis to measure the levels of IL-2. Figure 6C illustrates that Rv2029c-mediated TLR2/TLR12 activation reversed the BCG-induced reduction in MHC-II expression, and this effect was inhibited by blockage of TLR2/TLR12 signaling, indicating that Rv2029c enhanced the ability of BCG-infected macrophages to present antigens to T cells, as reflected by increased IL-2 levels in the co-culture supernatants of Rv2029c-preactivated macrophages infected with BCG and antigen-specific T cells (Figure 5D). These results demonstrate that Rv2029c promotes macrophage activation and antigen presentation in BCG-infected macrophages via TLR signaling.

### Rv2029c stimulates Th1-type T cell immunity and promotes effector/memory T cell proliferation

**Figure 7:**
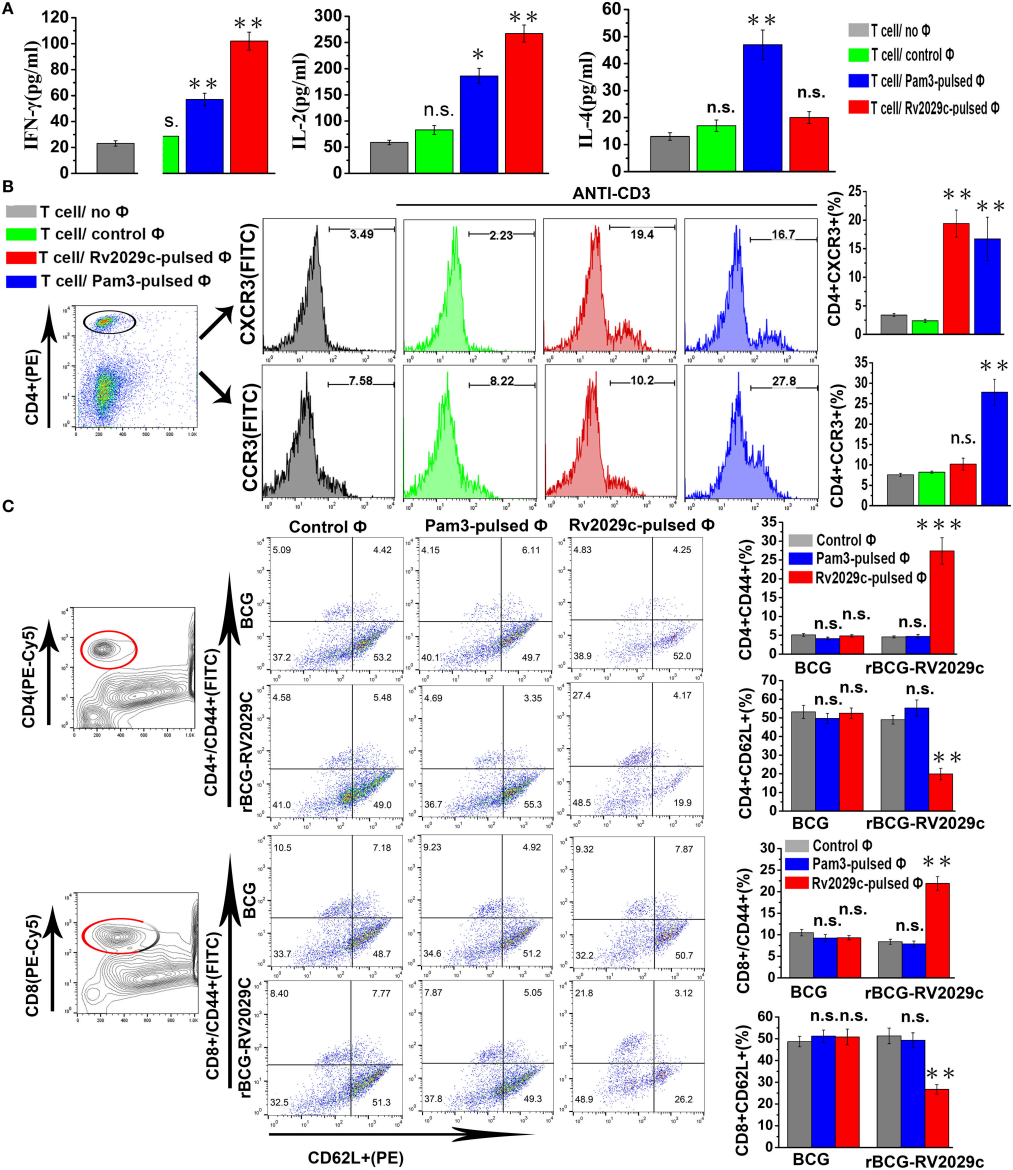
Rv2029c-activated macrophages stimulate Th1-type immune responses and induce effector/memory T cell proliferation. T cells were isolated from total mononuclear cells prepared from C57BL/6 mice immunized with PBS or Rv2029c (50 μg). Meanwhile, mouse peritoneal macrophages isolated from C57BL/6 mice were treated with R2029c (10 μg/ml) or Pam3 (5 μg/ml) for 24 h. The T cells and macrophages were then co-cultured at a ratio of 10:1 for 3 days at 37°C and pulsed with anti-CD3 (5 μg/ml) antibodies. Populations of T cells alone and T cells co-cultured with untreated macrophages served as controls. T cell proliferation was then assessed by flow cytometry. **(A)** Graphic depiction of the levels of IFN-γ, IL-2, and IL-4 in the culture supernatants from each group, as determined by ELISA analysis. **(B)** Semiquantitative RT-PCR **(B)** T cells collected during the experiment presented in **(A)** were stained with FITC-conjugated anti-CD4, PE-conjugated anti-CXCR3, or PE-conjugated anti-CCR3 monoclonal antibodies and analyzed by flow cytometry. Histograms and bar graphs indicate the numbers of CXCR3^+^ or CCR3^+^ T cells among the total population of R2029C-specific CD4+ T cells. **(C)** Splenocytes were isolated from C57BL/6 mice immunized with *Mycobacterium bovis* Bacillus Calmette-Guérin (BCG) or rBCG-Rv2029c, and macrophages were stimulated with Rv2029c (10 μg/ml), Pam3 (5 μg/ml), or PBS (control) for 24 h. mouse peritoneal macrophages were then washed and co-cultured with T cells at a ratio of 1:10 for 3 days. The splenocytes were stained with FITC-conjugated anti-CD4 monoclonal antibodies or FITC-conjugated anti-CD8, PE-conjugated anti-CD62L, and PE-Cy5-conjugated anti-CD44 monoclonal antibodies, and then analyzed by flow cytometry. The values shown represent the means ± standard deviations of the results obtained from three independent experiments. n.s., not significant; ^*^P < 0.05; ^**^P < 0.01.

Macrophages can efficiently prime naïve T cells to induce a Th1 response. To examine whether Rv2029c-enhanced MHC-II expression affects the ability of mouse macrophages to present antigens to T cells, we performed a Mixed Leukocyte Reaction (MLR) assay using Rv2029c-specific T cells co-cultured with Rv2029c-pulsed mouse peritoneal macrophages or mouse peritoneal macrophages alone. Rv2029c-specific T cells were incubated for 48 h with macrophages that were pretreated with Rv2029c, Pam_3_CKS_4_, or PBS, and the levels of IFN-γ, IL-2, and IL-4 in the supernatants were measured via ELISA. We found that T cells primed with Rv2029c-pulsed macrophages produced significantly higher levels of IFN-γ and IL-2 than T cells primed with untreated macrophages. Meanwhile, no significant changes in IL-4 secretion were detected between groups (Figure 7A).

Next, we evaluated the expression of the chemokine receptors CXCR3 and CCR3 via flow cytometry. T cells co-cultured with Rv2029c-pulsed mouse peritoneal macrophages exhibited significantly increased expression of CXCR3, but not CCR3, compared to the control group (Figure 7B). These findings suggest that Rv2029c-stimulated macrophages induce the development of T cells toward a Th1 phenotype. To then assess whether Rv2029c-activated macrophages are able to specifically stimulate CD4^+^ and CD8^+^ splenic T cell proliferation, splenocytes were isolated from C57BL/6 mice immunized with BCG or rBCG-Rv2029c, and mouse peritoneal macrophages were stimulated with Rv2029c (10 μg/ml), Pam_3_CKS_4_ (5 μg/ml), or PBS (control) for 24 h. The macrophages were then washed and co-cultured with T cells at a ratio of 1:10 for 3 days. The expression of CD62L and CD44 on CD4^+^ and CD8^+^ splenic T cells was analyzed by flow cytometry. As shown in Figure 7C, Rv2029c vaccination induced the formation and expansion of effector/memory T cells by significantly down-regulating CD62L and up-regulating CD44 expression on both CD4^+^ and CD8^+^ splenic T cells. Together, these findings indicate that Rv2029c induces the Th1 cell-mediated response and enhances the development of effector/memory T cells.

## Discussion

In this study, we demonstrate that boosting and immunotherapy with the multistage DNA vaccine A39 significantly increased the protective efficiency of the BCG vaccine, before and after exposure to MTB, respectively, compared to the control, as characterized by lower bacterial loads. The multistage A39 DNA vaccine was comprised of the early protective antigens Ag85A and Rv3425, and the latent antigen Rv2029c. Macrophages are optimally equipped to recognize pathogens and vaccines, and to simultaneously instruct the type, magnitude, and specificity of the subsequent immune responses (Gonzalez-Juarrero and O'Sullivan, 2011; Hmama et al., 2015). Previous studies have examined interactions between macrophages and the early antigens (Ag85A and Rv3425) (Russo et al., 2000; Xu et al., 2015); however, the precise mechanism underlying this protective immunity remains to be explained, but could provide a rationale for the clinical development of an A39 vaccine. Here we report that the latent antigen Rv2029c activated macrophages by increasing pro-inflammatory cytokine production, promoted macrophage apoptosis, and enhanced the expression of MHC-II molecules in BCG-infected macrophages in a TLR2/TLR12 dependent manner. Moreover, Rv2029c-treated macrophages activated naïve T cells, effectively polarized CD4^+^ and CD8^+^ T cells to secrete IFN-γ and IL-2, and specifically expanded a population of CD44^high^CD62L^low^CD4^+^/CD8^+^ effector/memory cells. Together, these data indicate that Rv2029c, as a specific recall antigen, contributes to the Th1 polarization of T cell immunity. Whether vaccination with the late antigen Rv2029c alone would be sufficient to induce a protective response is being researched in our lab, however, the multistage A39 DNA vaccine boosted the protective efficacy of BCG when administered both before and after MTB exposure.

Apoptosis is considered one of the most important host defense mechanisms against mycobacterial infection, as this process promotes killing of intracellular bacteria and processing of MTB antigens to induce proper immune responses (Behar et al., 2011). BCG-triggered apoptosis during infection leads to the release of MTB antigens in the form of apoptotic blebs, and the engulfment of these blebs by dendritic cells results in cross-priming, which effectively facilitates antigen presentation and culminates in the activation of T cells. In previous work, Farinacci et al. demonstrated that the recombinant BCG DeltaureC::hly(+) (rBCG) vaccine promotes macrophage apoptosis and improves the vaccine efficacy of rBCG, an effect that is dependent on enhanced cross-priming (Farinacci et al., 2012). Meanwhile, the early-secreted MTB antigen ESAT-6 promotes macrophage apoptosis and IFN-γ-induced MHC-II expression, but also exhibits strong immunotherapeutic potential as a component of a TB vaccine (Singh et al., 2005; Yu and Xie, 2012; Yang et al., 2015). Thus, BCG combined with antigen-induced apoptosis might comprise an effective strategy for the development of novel vaccines providing improved T cell stimulation and protection efficacy. In this context, our data showed that Rv2029c activated apoptosis-associated regulatory pathways in rBCG-Rv2029c—infected macrophages compared to those infected with BCG or rBCG-Bax, respectively. These results indicate that Rv2029c promotes the apoptosis of infected macrophages, potentially facilitating antigen presentation.

MHC-II molecules are continuously synthesized in response to infection, during which they are loaded with antigenic peptides in the MHC compartment and then exported to the plasma membrane, where they prime CD4^+^ T cells, mediating Th1 immunity to tuberculosis. MHC-II expression can be increased through the activation of macrophages, either with cytokines such as IFN-γ or via the activation of TLR signaling (Harding and Boom, 2010). Mycobacterial lipoproteins were found to suppress TLR2-dependent MHC-II expression in macrophages (Gehring et al., 2004). Conversely, TLR-2 (PGN), TLR3 [poly(I:C)], TLR4 (LPS), TLR-7, and TLR-9 ligands were reported to up-regulate MHC-II (Walseng et al., 2010; Bakhru et al., 2014). Thus, novel mycobacterial ligands for TLRs could serve as adjuvants in the development of tuberculosis vaccines. In the present study, we found that Rv2029c promoted TLR2 activation and MHC-II expression in mouse macrophages. TLR12 is intracellularly localized and plays a crucial role in the regulation of IL-12 production in response to parasitic infection (Raetz et al., 2013). We found that blockage of TLR12 attenuated the Rv2029c-induced increase in MHC-II expression observed in mice, and that Rv2029c bound specifically to TLR2, but not TLR12 (Figure S4), indicating that Rv2029c may modulate MHC-II expression via a TLR2-direct and TLR12-indirect manner.

Th1 lymphocytes are clearly involved in the early phase of host defense because they produce cytokines essential for neutrophil and monocyte recruitment to the site of infection. In particular, Th1 lymphocyte-induced IFN-γ promotes macrophage activation by stimulating phagosomal maturation and antigen presentation. Therefore, the induction and maintenance of Th1-polarized immune responses are thought to be critical to host protection against *M. tuberculosis* (Jasenosky et al., 2015). Indeed, defects in the IFN-γ signaling pathway were shown to lead to severe mycobacterial infections in both mice and humans (Andersen and Woodworth, 2014). IL-2 is secreted by Ag-activated CD4^+^ T cells during the primary immune response, and is clearly essential for the differentiation and expansion of naïve T cells into IFN-γ-producing effector cells in TB, indicating that continuous expression of IL-2 is required to confer resistance to TB progression (Uhlin et al., 2012; Clifford et al., 2015). In this study, naïve T cells primed with Rv2029c-treated macrophages produced significantly higher levels of IFN-γ than did those primed with untreated macrophages. Moreover, Rv2029c enhanced the ability of macrophages to present Rv2029c peptides to T cells, leading to increased IL-2 production. Conversely, this antigen had little effect on the levels of IL-4 secretion.

CXCR3 is primarily expressed on the surface of Th1- cells, and the cooperation of CXCR3 chemokines and ligands can lead to increased numbers and migration of Th1 lymphocytes; in contrast, CCR3 has been reported to play important roles in the Th2-cell homing pathway (Gangur et al., 2003; Brightling et al., 2005). Our results show that CD4^+^ T cells co-cultured with Rv2029c-treated macrophages showed significantly increased expression of CXCR3, but not CCR3, compared with those co-cultured with macrophages alone. Moreover, our results clearly demonstrate that Rv2029c-treated macrophages specifically induced the expansion of CD4/CD8CD44^high^CD62L^low^ memory T cells, which are capable of producing IFN-γ and are considered to be essential for acquired immunity and the efficacy of vaccines against TB (Gruppo et al., 2002). Thus, Rv2029c might comprise an ideal vaccine antigen, linking innate and adaptive immunity through macrophage activation by simultaneously inducing Th1-polarized T-cell expansion.

BCG infection suppressed MHC-II expression in macrophages that attenuated their ability to present peptides to T cells (Walseng et al., 2010). Thus, the variable protection provided by BCG could be due to an inherent inefficiency in MHC-II-dependent presentation of antigens to T cells by macrophages (Chen and Jensen, 2008). Activation of macrophages with ligands for TLR-9, 7, 5, 4, and 1/2 reversed the reduction in BCG-mediated MHC-II expression. BCG-triggered apoptosis *n* facilitates the release of antigens in the form of apoptotic blebs, resulting in the potential enhancement of antigen presentation. Therefore, MTB antigens can affect multiple intracellular events regulating MHC-II-dependent antigen presentation or apoptosis via TLRs, and might be necessary to achieve optimal delivery of BCG or other tuberculosis vaccines in their most immunogenic form (Bakhru et al., 2014). In this context, we found that Rv2029c-induced activation significantly enhanced MHC-II expression in BCG-infected macrophages at 4 h, and potentially promoted their ability to present peptides to T cells, as reflected by significantly higher levels of IL-2 production in T cells that were co-cultured with prior activation of BCG-infected macrophages induced by Rv2029c, compared with those co-cultured with BCG-infected macrophages alone. As is well-characterized, the surface levels of peptide-loaded MHC-II determine the efficacy of T cell activation, which in turn affects the levels of IL-2 secretion. We also observed that Rv2029c promoted apoptosis in BCG-infected macrophage at 48 h, which likely facilitated antigen presentation. The difference of the relative timings may account for Rv2029c- induced apoptosis and MHC II expression in BCG-infected macrophage, and the underlying causes need to be further explored.

In conclusion, latent antigen Rv2029c activated macrophages via TLR2/TLR12-mediated cross-talk among activated downstream pathways, providing a greater understanding of the interactions between latency-associated protein and the host immune system. Rv2029c challenge promoted apoptosis and reversed the reduction in MHC-II expression in BCG-infected macrophage via TLR, likely enhancing the ability of these cells to present antigens to CD4^+^ T cells and stimulated a Th1-type immune response. Additionally, when administered before and after MTB exposure, boosting with A39 conferred superior protective immunity, as characterized by efficient containment of late-stage infection, to vaccination with BCG alone, suggesting that Rv2029c-based TB vaccines could be utilized to control/delay reactivation or outbreak of active TB and/or reduce the rates of MTB transmission among the general populace.

## Author contributions

HS, HW, and YX conceived and designed the experiments; HS and SZ performed the experiments; HS and YX analyzed the data; HS and YX wrote the manuscript; HW and YX reviewed the manuscript and supervised the research. All authors read and approved the final manuscript.

### Conflict of interest statement

The authors declare that the research was conducted in the absence of any commercial or financial relationships that could be construed as a potential conflict of interest.
