# Supplementary material for: Mycobacterium tuberculosis Latent Antigen Rv2029c from the Multistage DNA Vaccine A39 Drives TH1 Responses via TLR-mediated Macrophage Activation
Source: Front Microbiol. 2017 Nov 17;8:2266. doi: 10.3389/fmicb.2017.02266 (PMC5698697; doi:10.3389/fmicb.2017.02266)
Supplement: Supplementary file 4 [file Image1.PDF]

Mycobacterium tuberculosis Latent Antigen Rv2029c from the Multistage DNA Vaccine A39 Drives TH1 Responses via TLR-mediated Macrophage Activation

Haibo Su[1, 2, 3#], Shenglin Zhu[1#], Lin Zhu[1], Cong Kong[1], Qi Huang[1], Zhi Zhang[3],Honghai Wang[1],

Ying Xu[1]*

[1]State Key Laboratory of Genetic Engineering, Institute of Genetics, School of Life Science, Fudan University, No. 220 Handan Road, Shanghai 200433, China

[2]GMU-GIBH Joint School of Life Science, Guangzhou Medical University, No. 195 Dongfengxi Road, Guangzhou, 510000, China

[3]The second people's hospital of Guangdong province, No. 466 Xingang Road, Guangzhou, 510220, China

Keywords: Latent antigens, *Mycobacterium tuberculosis*, Macrophages, Tuberculosis, TB vaccine.

[#]The authors contributed equally to this work.

***Corresponding authors**: Y. Xu

E-mail: yingxu2520@fudan.edu.cn; Tel: +86 21 5163 0587; Fax: +86 21 5163 0587

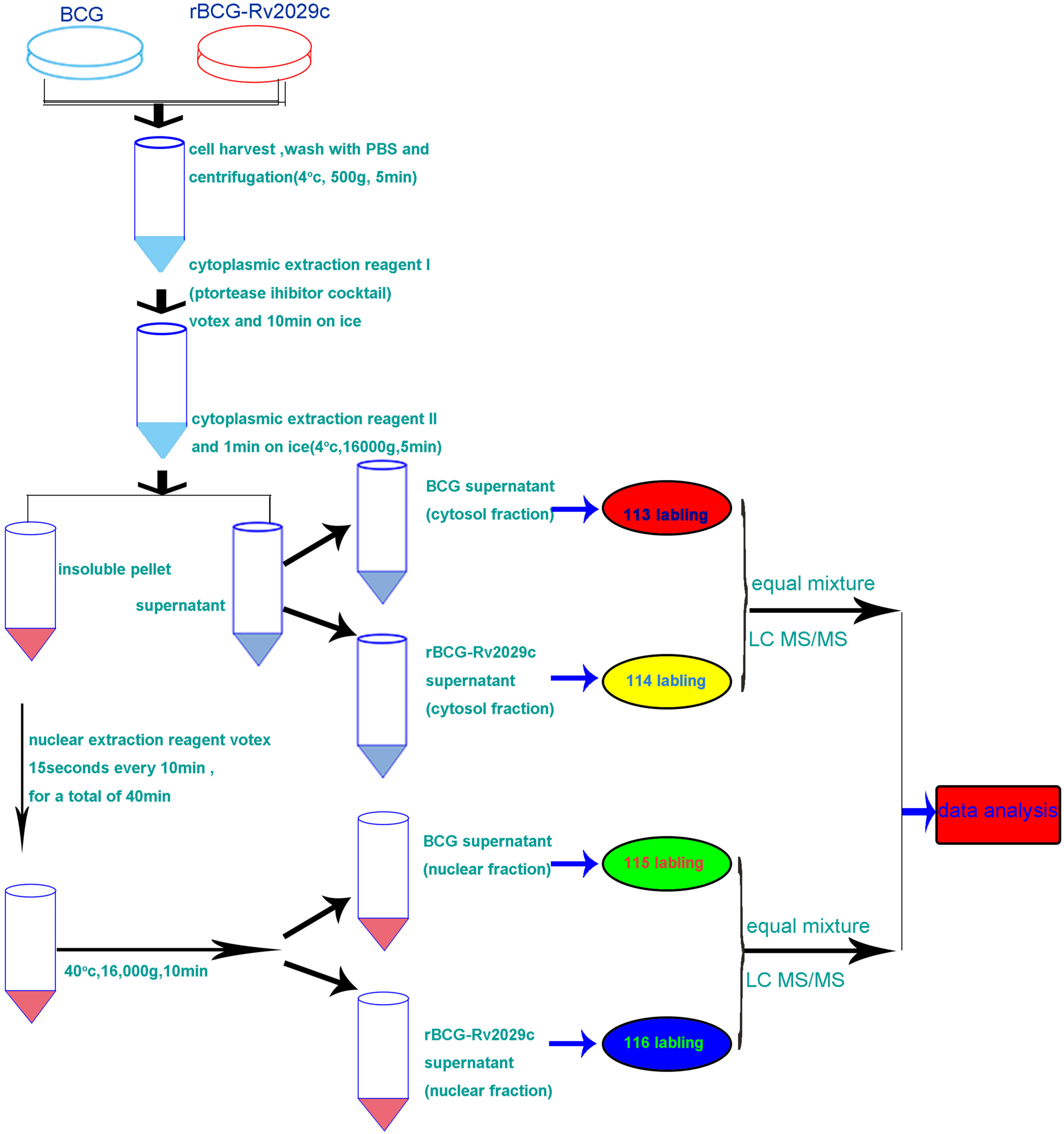

Supplementary Figure S1. Schematic diagram describing the iTRAQ quantitative proteomic approach used for protein extraction and identification.

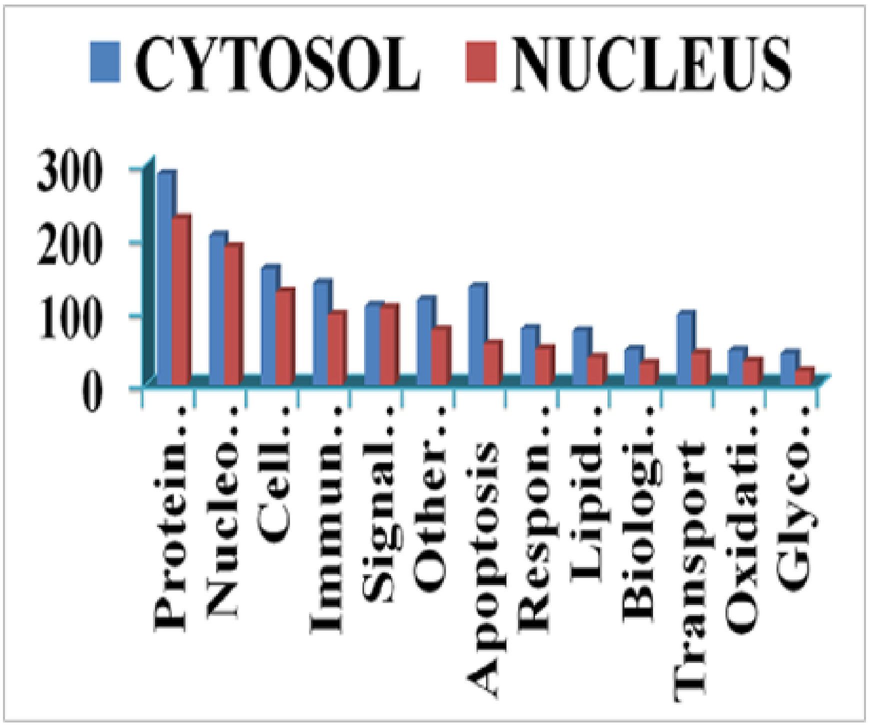

Supplementary Figure S2. Molecular functions of the differentially expressed proteins identified in Rv2029c-stimulated macrophages. Proteins showing an H/L higher than 1.2 or less than 0.8 in the nucleus and cytosol, respectively, were submitted to PANTHER (http:// www.pantherdb.org/) to obtain information regarding their known functions.

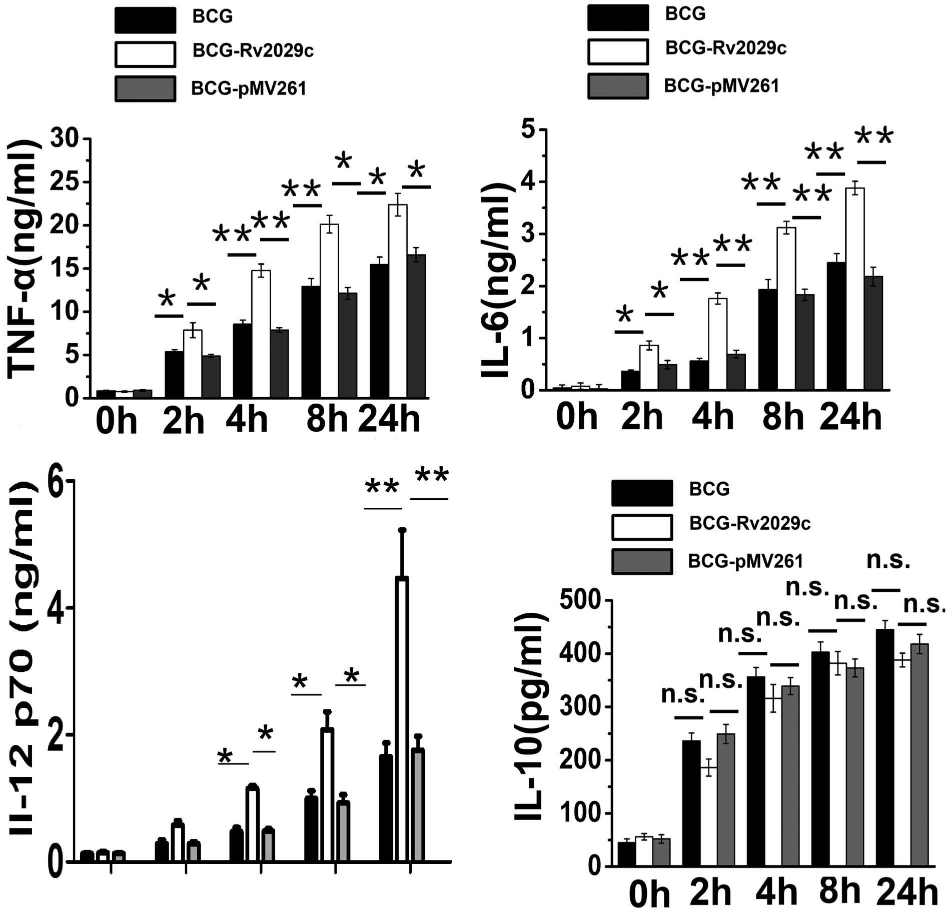

Supplementary Figure S3. ELISA analysis of TNF-α, IL-6, and IL-12 p70, and IL-10 production in RAW264.7 cells infected for 0-24 h with BCG, rBCG-pMV261, or rBCG-Rv2029c.

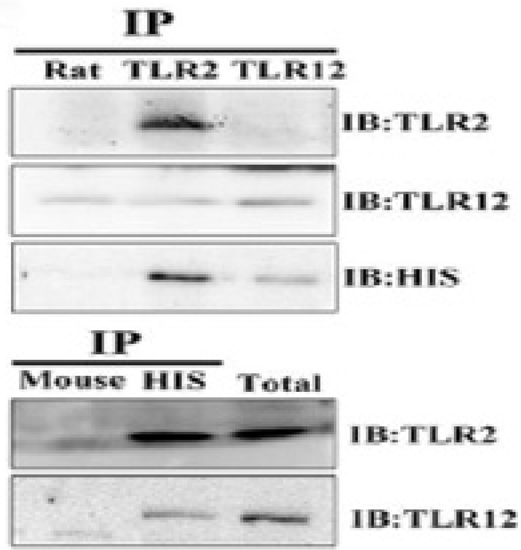

Supplementary Figure S4. Rv2029c interacts with TLR2, but not TLR12. RAW264.7 cells were treated with Rv2029c for 8 h, lysed, and subjected to immunoprecipitation using anti-rat Ig G, anti-mouse Ig G, anti-His, anti-TLR2, or anti-TLR12 antibodies. Precipitates were separated by SDS-PAGE and proteins were visualized by immunoblotting with anti-His, anti-TLR2, or anti-TLR12 antibodies. The plot shown is representative of the results obtained from two independent experiments.

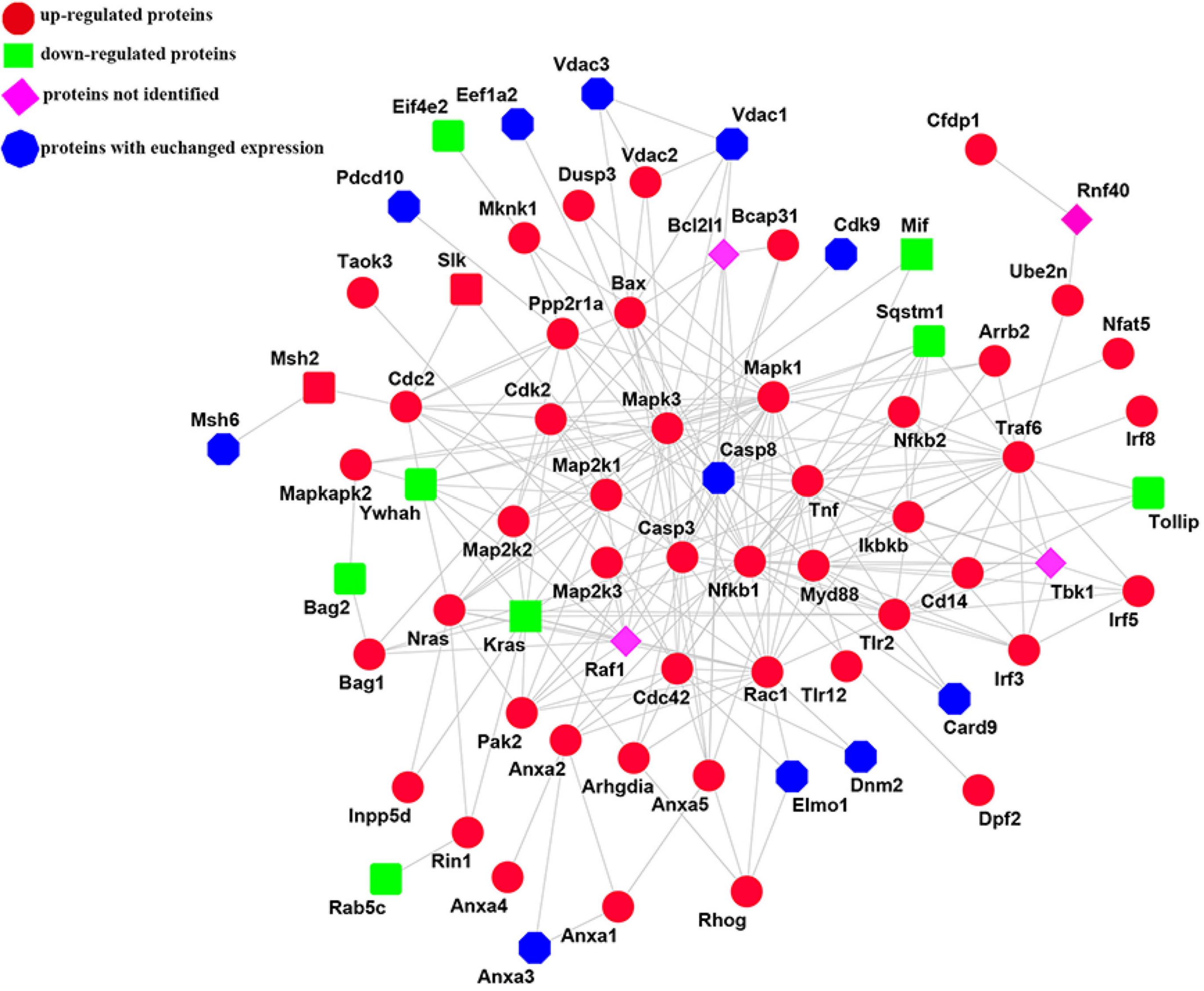

Supplementary Figure S5. Data-dependent reconstructed network illustrating how the activated signaling pathways coordinate apoptosis balance. The Rv2029c-dependent differentially expressed proteins identified in our study were submitted to STRING (http://string.embl.de/) for network construction. The resulting network links were edited using Cytospace 3.1.0 software.
